# Improving the touchscreen-based food approach-avoidance task: remediated block-order effects and initial findings regarding validity

**DOI:** 10.12688/openreseurope.13241.1

**Published:** 2021-03-24

**Authors:** Hannah van Alebeek, Sercan Kahveci, Jens Blechert

**Affiliations:** 1 Department of Psychology, Paris-Lodron-University of Salzburg, Salzburg, Austria; 2 Center for Cognitive Neuroscience, Paris-Lodron-University of Salzburg, Salzburg, Austria

**Keywords:** approach-avoidance task, implicit association task, food, external eating, touchscreen, reliability

## Abstract

Approach biases to foods may explain why food consumption often diverges from deliberate dietary intentions. When cognitive resources are depleted, implicit responses may contribute to overeating and overweight. Yet, the assessment of behavioural biases with the approach-avoidance tasks (AAT) is often unreliable. We previously addressed methodological limitations of the AAT by employing naturalistic approach and avoidance movements on a touchscreen (hand-AAT) and instructing participants to respond based on the food/non-food distinction. In the consistent block, participants were instructed to approach food and avoid objects while in the inconsistent block, participants were instructed to avoid foods and approach objects. Biases were highly reliable but affected by the order in which participants received the two task blocks. In the current study, we aimed to resolve the block order effects by increasing the number of blocks from two to six and validate the hand-AAT with the implicit association task (IAT) and self-reported eating behaviours. We replicated the presence of reliable approach biases to foods and further showed that these were not affected by block order. Evidence for validity was mixed: biases correlated positively with external eating, food craving and aggregated image valence ratings but not with within-participants differences in desire to eat ratings of the images or the IAT. We conclude that hand-AAT can reliably assess approach biases to foods that are relevant to self-reported eating patterns and were not probably confounded by block-order effects.

## Introduction

Habitual behaviours like eating rely primarily on their implicit association with environmental cues and little on deliberate intentions ( van't Riet *et al.*, 2011). As such, implicit processes may help to explain why some people fail to follow their diet-related intentions. Stronger implicit approach bias toward food has been related to higher food intake when self-regulatory capacity is low and to more uncontrolled eating in impulsive individuals ( Booth *et al.*, 2018; Kakoschke *et al.*, 2015; Kakoschke *et al.*, 2017b). Yet, individuals with clinically diagnosed binge eating do not show an increased approach bias to food compared to healthy controls ( Paslakis *et al.*, 2017), but at the other end of the spectrum, a decrease or absence of approach bias towards food may help to explain the persistently reduced food intake in individuals with anorexia nervosa ( Neimeijer *et al.*, 2019; Paslakis *et al*., 2016; for a review see Paslakis *et al.*, 2020). Perhaps most importantly, a recent review has concluded that modification of approach bias can help reduce food consumption, thereby supporting a causal role for approach within normal and disordered food consumption ( Kakoschke *et al.*, 2017a). Hence, research has begun to focus on the reliable assessment of implicit processes so they can be measured and targeted for treatment in uncontrolled eaters.

On a behavioural level, implicit responses to food cues can be quantified using the approach-avoidance task (AAT). In the AAT, participants are required to approach and avoid two stimulus categories. An approach bias is inferred if a stimulus category, such as food, is approached faster than avoided, and this advantage for approach is larger than that of another stimulus category, such as office articles ( Lender *et al.*, 2018). Two different task instructions have been used: in the irrelevant-feature AAT, participants must approach or avoid stimuli based on a feature of the stimulus that is unrelated to the bias being measured (e.g. frame tilt or frame colour), while in the relevant-feature AAT, the participants must approach or avoid based on the stimulus category, thereby directing attention to the food/non-food distinction. Notably, relevant-feature AATs have yielded reliable approach biases to food, whereas biases in irrelevant-feature AATs were absent or unreliable ( Lender *et al*., 2018; Meule *et al.*, 2019). Next to instruction, different task set-ups affect approach bias: e.g., in set-ups using a joystick, approach bias effects are elicited primarily by the stimuli zooming in or out ( Krieglmeyer & Deutsch, 2010; Rinck & Becker, 2007) and accordingly, approach bias could be attained when stimuli were zoomed with simple key presses ( Becker *et al.*, 2015; Peeters *et al*., 2012). However, it was shown that manipulating a stimulus’ position at a distance (as simulated with the zoom-feature) elicits smaller approach biases than moving oneself to approach and avoid a stimulus ( Rougier *et al*., 2018). Suboptimal task set-ups as well as the use of irrelevant-feature instructions may explain why some studies do not report approach biases to foods or do not report correlations with self-reported eating behaviours ( Matheson, 2018; Meule *et al*., 2020) while others do ( Brockmeyer *et al.*, 2015; Lender *et al*., 2018; Maas *et al*., 2017a; Meule *et al*., 2019).

To improve the assessment of approach bias, we developed a new variant of the AAT. In the hand-AAT, participants slide their hand toward or away from a picture on a touchscreen, with movement direction depending on the stimulus category (relevant-feature: food vs. object). On a positive note, this task set-up yielded reliable approach biases to foods that correlated with explicit desire ratings in a healthy student population ( Kahveci *et al.*, submitted), but on a more negative note, the order of instruction blocks confounded interindividual differences in approach bias. While being more reliable than irrelevant-feature AATs ( Phaf *et al.*, 2014), feature-relevant AATs require at least one instruction switch: participants have to approach food and avoid objects in the consistent block, but avoid foods and approach objects in the inconsistent block. We showed in the hand-AAT, as well as in three other feature-relevant AATs ( Kahveci *et al*., submitted; Wittekind *et al*., submitted), that approach bias to food and their correlations with food craving are larger for participants starting with the inconsistent block.

In the current study, we thus sought to minimize block order effects in the hand-AAT by using six blocks rather than the usual two. We also aimed to replicate the finding that stimulus-specific desire ratings predict approach bias for those stimuli and we included the single category implicit association task (IAT) with approach and avoidance words to validate the task with an implicit measure of approach associations. As preregistered ( https://osf.io/ez7ka/), we expected that the updated hand-AAT would reliably detect behavioural approach bias to foods relative to objects. We further hypothesized that more desired food stimuli would be approached faster and avoided slower than less desired food stimuli ( Kahveci *et al*., submitted), and that participants with a stronger AAT approach bias would show stronger approach associations in the IAT as well as higher levels of self-reported food craving. Lastly, we explored relationships between approach biases and implicit approach associations on the one hand and external eating, restrained eating, body mass index (BMI), and mean ratings of the food pictures on the other hand.

## Methods

### Participants

We recruited 59 students (24 male) of the University of Salzburg via announcements during lectures and by posting flyers on social media platforms. Subsequent to the online-questionnaires, two participants cancelled their appointment and one did not show up to the lab-session. As preregistered, 10 participants were excluded because they had an average desire-to-eat rating below 30 or above 70 and three more were excluded due to an excessive outlier or error rate on the AAT (>15%). Our final sample included 43 participants (17 male), aged between 18 and 30 years (mean [ *M*] = 22.95, standard deviation [ *SD*] = 3.54), and with a BMI between 18.02 and 39.67 kg/m² ( *M* = 23.13, *SD* = 4.54). Participants’ orientation towards healthy ( *M* = 4.59, *SD* = 1.04) and natural ( *M* = 4.06, *SD* = 1.31) foods, as assessed with the eating motivation scale (TEMS) ( Renner *et al.*, 2012), did not differ from the health (Welch’s *t* (45) = 0.74, *p* = .462) and natural orientation (Welch’s *t* (45) = .54, *p* = .593) of the population.

### Questionnaires

Reliability values are based on the full sample. As Cronbach’s α systematically underestimates reliability, we additionally report McDonald’s ω ( Mcdonald, 1978; Revelle & Zinbarg, 2009; Sijtsma, 2009).

***TEMS – natural concern and health motivation.*** TEMS was used to compare this sample’s orientation towards natural and healthy food, compared to the general population. Reliability was good for the natural concern (α = .92, ω = .92) and health motivation subscale (α = .86, ω = .87).

***Food craving questionnaire – state (FCQ-S) and trait (FCQ-T-r).*** The German versions of the FCQ-S and FCQ-T-r ( Meule *et al.*, 2014; Meule *et al.*, 2012a) were used to measure state and trait food craving, respectively. Both had excellent reliability in this study (α = .90, ω = .90).

***Dutch eating behavior questionnaire (DEBQ).*** The three subscales of the DEBQ ( Van Strien *et al.*, 1986) were used to measure emotional eating, external eating, and restrained eating. All three subscales were reliable (emotional eating: α = .92, ω = .92; external eating: α = .87, ω = .86; restrained eating: α = .86, ω = .85).

***Other scales.*** The perceived self-regulatory success in dieting scale ( Meule *et al.*, 2012b) and the positive and negative affect schedule ( Watson *et al.*, 1988) were administered but not analysed.

### Materials and apparatus

The AAT was administered using a 23-inch iiyama ProLite T2336MSC-B2 touchscreen monitor with a resolution of 1920 × 1080 pixels, placed in portrait-format with a 10% tilt towards the participant.

The AAT included 24 object and 24 food images, selected from the food-pics_extended database ( Blechert *et al.*, 2019) and the FRIDa database ( Foroni *et al.*, 2013). The food images were drawn semi-randomly for each participant from a larger pool of 60 individually rated food items ^1^ to ensure an equal number of desired and non-desired foods. The IAT used the 12 most highly desired stimuli of the personalized stimulus set used in the AAT.

**Figure 1.**
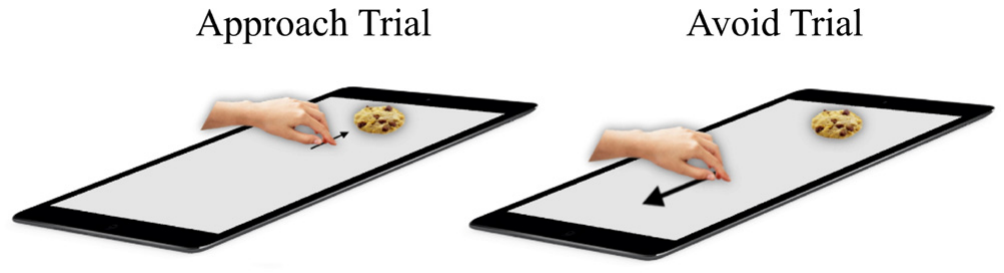
Hand-AAT. On approach trials, the participant slides their hand from the middle towards the food/object and on avoid trials the participants slides their hand from the middle in the direction opposite to the food/object stimulus.

***AAT.*** In a typical AAT trial, participants placed their hand on a symbol centrally on the screen, and after a random delay between 300ms and 700ms, a stimulus was displayed on the distal side of the touchscreen. Participants approached or avoided the stimulus by sliding their hand towards it or away from it, respectively ( Figure 1). After approaching a stimulus, it ‘snapped’ to the hand and was moved back to the center of the screen along with the hand. Stimuli were avoided by moving the hand away from the stimulus and towards an avoidance zone at the proximal side of the touchscreen. After avoiding a stimulus, the stimulus disappeared. Participants completed a 12-trial practice block, followed by six blocks with 48 trials each. At the start of each block, participants were instructed to either approach foods and avoid objects (consistent blocks), or to avoid foods and approach objects (inconsistent blocks). This alternated from one block to the next and the order was counterbalanced between participants. Stimuli were shown in semi-random order to ensure each stimulus category was not repeated more than thrice ( Wiers *et al.*, 2010). An error was recorded if participants lifted their hand or initiated a movement in the wrong direction. The time from stimulus onset until movement onset was chosen as the reaction time (RT) measure.

***Single-category implicit association task (IAT).*** During the IAT, participants sorted 6 German approach words (e.g., ‘approach’, ‘grab’, ‘…’), 6 avoidance words (e.g., ‘avoid’, ‘remove’, ‘…’), and 12 food images into categories displayed at either side of the screen using the E and I keys. “Approach” was always displayed at one side, and “Avoidance” at the other; this was counterbalanced by the participant. Additionally, “Food” was displayed either at the left or the right during the testing blocks, alternating between blocks and counterbalanced by participant in accordance with the AAT, such that participants received the same block order in both tasks.

During the first 24 practice trials, participants only sorted approach/avoidance related words; during the two subsequent testing blocks, participants sorted these words as well as food images. Each block consisted of 84 trials, of which 24 were food trials, 24 were words to be categorized on the same side as the food images, and 36 were words to be categorized on the other side. This unequal division was required to be able to balance the number of responses on either side, while having two stimulus categories on one side and one on the other side ( Karpinski & Steinman, 2006).

### Procedure

The study was conducted with permission granted by the ethics committee of the Paris-Lodron University of Salzburg (EK-GZ: 27/2018), in accordance with the Declaration of Helsinki and participants provided written consent to study procedures. Prior to the start of the study, participants were instructed to fast for at least four hours, with the intent of increasing their food cravings. After these four hours, they completed online-versions of the FCQ-T-r, FCQ-S, TEMS and DEBQ, and rated all food and object stimuli on valence, and all food stimuli on desire-to-eat. Exactly one week after this online-session, participants fasted again for at least four hours and were then invited to the lab. Here they completed the FCQ-S, followed by the AAT, and the FCQ-S again, afterwards their height was measured, the IAT was administered, their weight was measured, and they were reimbursed after signing a form of consent.

### Data processing

Data were processed and analyzed as pre-registered. First, RTs were excluded if they were above 1500ms or below 200ms, or if the response was incorrect; then, RTs were square-root transformed to improve normality; after this, RTs were excluded if they deviated more than 3 *SD*s from the participant’s mean.

For the multilevel analyses, we included all level 1 fixed effects also as random effects nested under stimulus, and we further included random intercepts per stimulus and random slopes for trial number per block per subject. Significance of highest-order model terms was tested by comparing a model with the effect to a model without the effect using a Wald chi-square test. The reported standardized regression coefficients are based on the full model.

For the computation of AAT and IAT D-scores, all RTs below 10s were included and error trials were replaced by the correct block mean plus a 600ms penalty. D-scores were computed by subtracting the mean RT for each consistent block from the mean RT of the adjacent inconsistent block, dividing the result by the standard deviation of the two involved blocks, and averaging the D-scores of all sets of two blocks to result in a final D-score ( Greenwald *et al.*, 2003). The IAT D-score constitutes the association bias and the AAT D-score constitutes the behavioural approach bias.

## Results

### Reliability

Bootstrapped split-half reliability was computed using the AATtools package ( Kahveci, 2020) for R ( R Core Team, 2019). The sample was split randomly, outliers were excluded, and bias scores were computed in accordance with the Methods section, and scores from both halves were correlated. This process was repeated 10000 times and the resulting split-half correlations were averaged and corrected for halved test length. The AAT was reasonably reliable for an implicit measure, *r*_SB_ = .64, as was the IAT, *r*_SB_ = .66.

### Bias

**Figure 2.**
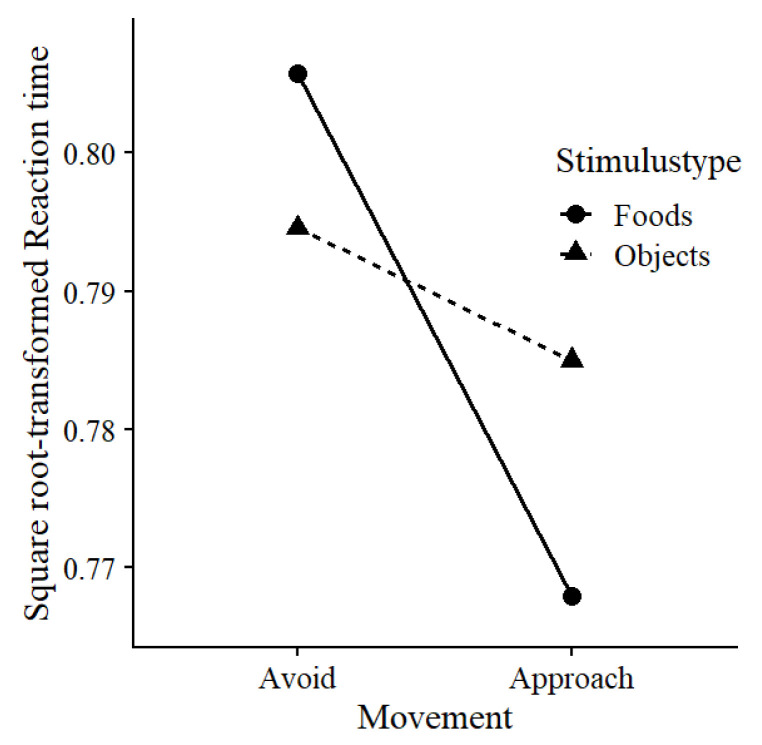
Behavioural approach bias to foods. Mean reaction times in seconds per condition.

We examined whether there was a greater behavioural approach bias for foods compared to objects. We predicted square root-transformed RTs using fixed and random factors for Movement (0 = avoid, 1 = approach) and Stimulustype (0 = object, 1 = food), as well as random intercepts per stimulus and random slopes of trial number per block per participant, as described in Equation 1. Movement and Stimulustype interacted, χ² (1) = 21.20, *p* < .001, *β* = -.128. Follow-up analyses confirmed that, compared to objects, foods were avoided slower, χ² (1) = 6.63, *p* = .010, *β* = .057, ΔRT = 16ms, and approached faster, χ² (1) = 18.00, *p* < .001, *β* = -.095, ΔRT = 28ms ( Figure 2). 

            
sqrtRT ~ Movement * Stimulustype + (Movement * Stimulustype | Subject) + (1 | Stimulus) + (TrialNumber – 1 | Subject/Block)   (1)

As for the IAT, D-scores significantly differed from zero, indicating an association between food and approach, *t* (42) = 3.00, *p* = .003. There was no significant relationship between behavioural approach bias for highly desired stimuli and implicit associations for highly desired stimuli, *r* (41) = -.12, *p* = .446.

### Desire

To investigate the effect of the participant’s desire to eat specific foods on behavioural approach bias, we predicted square root-transformed RTs with movement, desire, and their interaction, as fixed and random effects, as well as random intercepts per stimulus and random slopes but no intercepts for trial number per block, as depicted in Equation 2. There was no larger difference between approach and avoidance reaction times for stimuli that were more desired, χ ^2^ (1) = .87, *p* = .350, *β* = .028.

            
sqrtRT ~ Movement * Desire + (Movement * Desire | Subject) + (1 | Stimulus) + (TrialNumber – 1 | Subject/Block)                     (2)

### Craving, BMI, and eating behaviour

**Figure 3.**
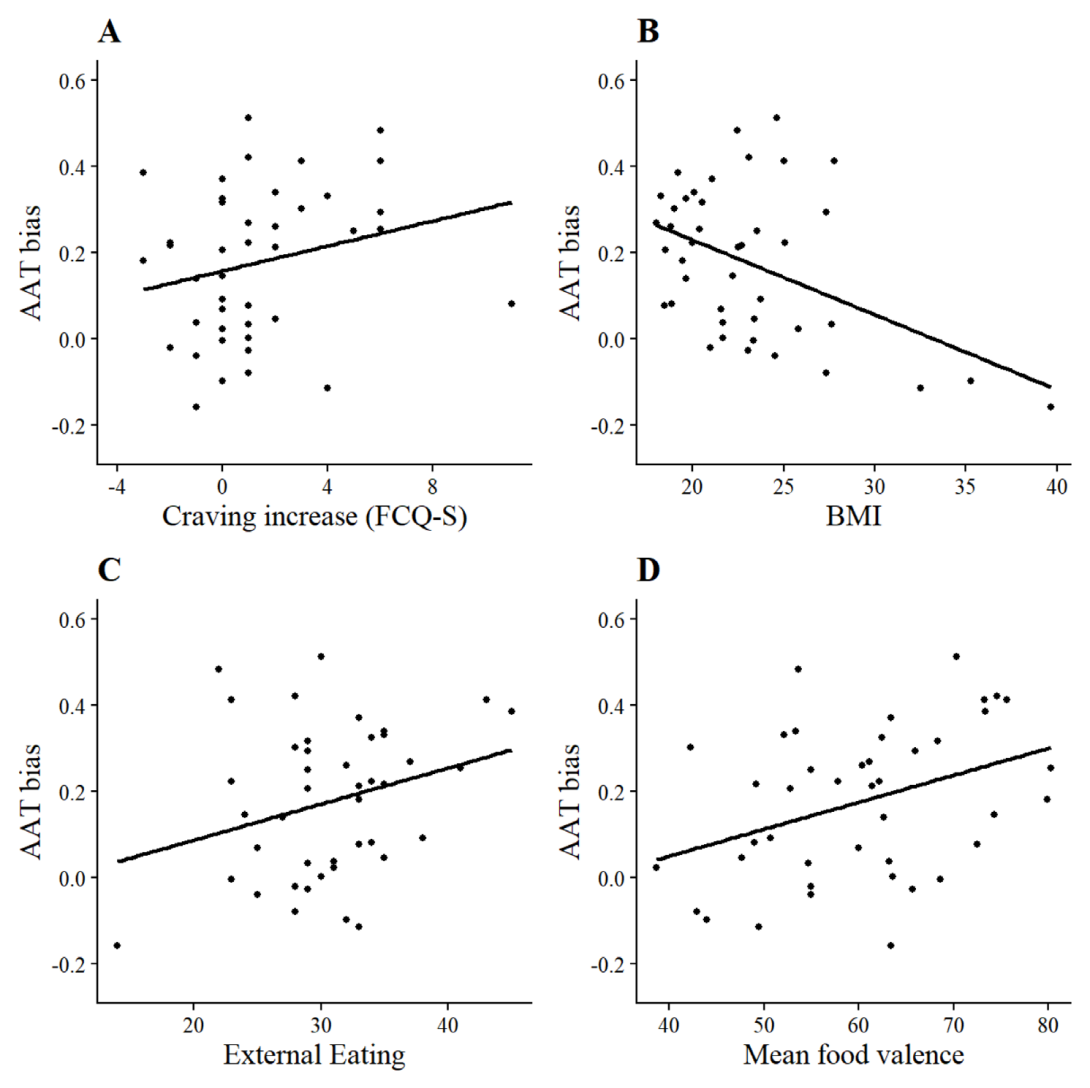
Scatterplots of significant correlations between AAT bias and **a**) increase of the FCQ-S from pre to post-AAT, **b**) body mass index, **c**) DEBQ-external eating and **d**) mean food valence.

We explored correlations between AAT and IAT D-scores on the one hand, and the DEBQ subscales, state and trait food craving, BMI and mean ratings of the foods on the other hand. Correlations are listed in Table 1. Higher external eating scores related to higher AAT approach bias and IAT association bias scores. AAT bias correlated positively with the increase in craving from pre-test to post-test and with mean ratings of food valence, but negatively with BMI ( Figure 3). The latter effect must be interpreted carefully, as only three participants with obesity (BMI > 30) were included in the sample, and the correlation was non-significant ( *r* (38) = -.22, *p* = .177) after those participants were excluded. It should also be noted that power to detect a medium correlation ( *r* = .3) was suboptimal (1 - β = .51), which may have obscured true effects while moving spurious effects to the foreground. 

**Table 1.** Correlations between AAT and IAT D-Scores on the one hand and self-reports on the other hand. *significance at trend-level (α < .1).

	AAT bias		IAT bias	
	*r* (41)	*p*	*r* (41)	*p*
Pre-AAT craving (FCQ-S)	.05	.733	.16	.292
Post-AAT / Pre-IAT craving (FCQ-S)	.23	.143	.13	.401
Δ Craving increase (FCQ-S)	**.31**	**.040**	-.03	.858
Trait craving (FCQ-T-r)	.02	.894	.13	.389
Body Mass Index	**-.45**	**.002**	.19	.225
External eating (DEBQ)	**.37**	**.016**	**.32**	**.036**
Restrained eating (DEBQ)	-.06	.684	.29 *	.057
Mean food valence	**.38**	**.012**	-.28 *	.074
Mean food desire	.30 *	.052	.02	.900

### Block order effects

We explored whether the demonstrated behavioural approach bias of the AAT was affected by whether participants received the consistent or inconsistent block first. We predicted square root-transformed RTs using fixed and random effects for Movement, Stimulustype, and their interactions, fixed effects for Order and its interactions with the other fixed effects, as well as random intercepts per stimulus and random slopes but no intercepts for trial number per block, as described in Equation 3. Block order did not affect the approach bias toward foods, χ ^2^ (1) = 3.30, *p* = .069, *β* = .063.

            
sqrtRT ~ Movement * Stimulustype * Order + (Movement * Stimulustype | Subject) + (1 | Stimulus) + (TrialNumber-1 | Subject/Block)   (3)

Accordingly, D-scores ( *M* = .17, *SD* = .16) for participants starting with the inconsistent block did not differ significantly from D-scores ( *M* = .16, *SD* = .22) for participants starting with the consistent block (Welch’s *t* (32) = .3, *p* = .80). As for the IAT, approach associations did not differ significantly for block order (Welch’s *t* (40) = 1, *p* = .20).

## Discussion

We found that participants had a behavioural approach bias toward food in the AAT and implicit approach associations with food in the IAT. Both biases were stronger in individuals with higher external eating. Larger AAT biases were further found in participants giving overall higher mean food valence ratings, and those reporting increases in food craving over the experiment. We thus demonstrated a relationship between forms of cue-reactivity in the domains of implicit approach responses, craving, and self-reported patterns of eating behaviour. Yet, individually more desired food items did not show evidence of larger AAT biases than individually less liked food items, and the AAT and IAT did not correlate with each other, or with state craving, trait craving, restrained eating, or desire to eat different foods despite comparable reliability.

The lack of an association between AAT and IAT scores is not an uncommon finding in the eating literature ( Maas *et al*., 2017a; Woud *et al.*, 2016) and in implicit bias research more broadly ( Pieters *et al.*, 2014), with some researchers even finding a negative correlation between the two ( Larsen *et al*., 2014; Warschburger *et al.*, 2018). These findings underline that the two tasks measure different concepts: the AAT measures the readiness to perform approach and avoidance movements in response to a stimulus, while the approach-avoidance IAT measures associations between the stimulus and the cognitive concepts of approach and avoidance – associations that do not necessarily overlap with actual behavioural tendencies. Despite being unrelated to each other in the current study, both tasks were associated with external eating, the tendency to eat in response to external cues rather than internal ones such as hunger. This suggests that some participants may display external eating due to strong cue-elicited approach responses, while others may display external eating due to a more cognitive association between food and consumption, for example due to food-related beliefs and cultural norms.

BMI was negatively related to AAT approach bias in our study. However, this effect depended on the inclusion of the three participants with a BMI over 30, and it is contrary to most approach-avoidance studies involving obese participants, which find that obesity is related to *higher* approach bias towards food ( Mehl *et al.*, 2018). The literature also reports a positive relationship between IAT food-approach associations and obesity, which we did not replicate, thus supporting the interpretation that the current results were far too underpowered to reveal any obesity-related effects ( Kemps & Tiggemann, 2015; Maas *et al*., 2017b).

We could not replicate the finding that interpersonal differences in the desire to eat individual food items predict approach bias for those individual food items ( Kahveci *et al.*, 2020; Kahveci *et al*., submitted). This may be due to the one-week delay between the desire to eat ratings and approach bias measurement in this study – the aforementioned studies collected ratings directly after measurement of approach bias. The relationship between approach bias and food preferences are thus likely to be momentary, as the desire for specific foods changes within days ( Reichenberger *et al*., 2018) and also IAT-consumption behaviour relationships have been shown only in states of high momentary state craving and hunger ( Richard *et al.*, 2019).

On a more positive note, we successfully remedied the confounding effect of block order on approach bias scores, which was found in the previous feature-relevant AATs ( Kahveci *et al*., submitted; Wittekind *et al*., submitted): biases were found regardless of whether the inconsistent or consistent block started the block sequence. This is likely because we increased the number of blocks to six, which lessened the temporal primacy of one condition over another. Block order effects introduce differences in participants’ bias scores which are unrelated to the participant’s inherent approach bias, and thus reduce the correlations between the measured bias and external measures (e.g. for assessing validity). Therefore, we recommend increasing the number of blocks in the AAT in future research to decrease the artificial differences in behavioural approach bias when it is not feasible to avoid counterbalancing block order.

Reliability of the hand-AAT may seem promising when considering the ‘reliability crisis’ in the broader field of cognitive bias measurement ( LeBel & Paunonen, 2011; McNally, 2019). However, it is not uncommon that feature-relevant AATs attain reliability estimates in the upper range across implicit measures ( Gawronski *et al.*, 2011) and reliability in current task set-up was lower than in our previous version of the hand-AAT ( Kahveci *et al*., submitted). Critically, higher reliability in this previous study may be partly explained by block-order effects, which likely increased the range of approach bias scores, as well as by less variance in stimulus valence, which likely lowered RT variability within the participants. The reliability of the current paradigm and its currently suboptimal power, may be improved by increasing the number of trials or by standardizing stimulus sets with respect to their graspability ( Baker *et al*., 2020). As the task has relationships to cue reactivity, lacks block order effects, and has a reliability slightly under what is considered sufficient in psychometric theory (r = .7; Rammstedt, 2004), it may serve as a good starting point for future research in the measurement and modification of automatically triggered appetitive responses as they occur in habitual behaviours.

## Data availability

OSF: Improving the touchscreen-based food approach-avoidance task: remediated block-order effects and initial findings regarding validity 

https://doi.org/10.17605/OSF.IO/EZ7KA ( Kahveci *et al*., 2021)

This project contains the following underlying data:

Raw data files:

- Anthropometry.sav (height and weight measured during the lab session)
- 1_SCIAT.csv – 61_SCIAT.csv (separate IAT files for each participant)
- 1_2019-11-07-17-25.csv - 61_2020-02-04-16-16.csv (separate AAT files for each participant)
- home.sav (demographics, FCQ-T-r, the TEMS, perceived self-regulatory success in dieting scale, DEBQ, FCQ-S and individual stimulus rating on valence and desire to eat assessed during the online survey)
- post.sav (the FCQ-S administered subsequent to the AAT)
- pre.sav (the positive and negative affect schedule and the FCQ-S administered prior to the AAT)

Pre-processed data files:

- HandSRT2_IAT_longformart.csv (trial-level IAT data for all participants)
- HandSRT2_preppeddata.csv (trial-level AAT data for all participants)
- HandSRT2_masterfile.csv (participant-level questionnaire sum scores and aggregated AAT as well as IAT scores)

Analyses and pre-processing scripts:

- IATextraction.R (R-code to merge and pre-process IAT data as well as to compute IAT D-scores)
- Datapreparation.R (R-code to merge and pre-process AAT and questionnaire data and to subsequently combine them with the pre-processed IAT data)
- Analyses.R (R-code used for analyses of results)

Data are available under the terms of the Creative Commons Zero "No rights reserved" data waiver (CC0 1.0 Public domain dedication).

## Footnotes

^1^ Indexes of food items in the food-pics_extended database: 0004, 0060, 0062, 0110, 0111, 0113, 0131, 0134, 0169, 0173, 0180, 0186, 0187, 0192, 0244, 0194, 0196, 0197, 0199, 0201, 0214, 0221, 0234, 0263, 0266, 0267, 0282, 0283, 0226, 0317, 0325, 0361, 0366, 0380, 0394, 0396, 0397, 0399, 0424, 0439, 0438, 0467, 0510, 0515, 0539, 0654, 0682, 0715, 0742, 0759, 0811, 0800, 0809, 0818, 0819, 0840, 0860, 0874, 0880.
