# Improving the touchscreen-based food approach-avoidance task: remediated block-order effects and initial findings regarding validity

**DOI:** 10.12688/openreseurope.13241.3

**Published:** 2021-08-20

**Authors:** Hannah van Alebeek, Sercan Kahveci, Jens Blechert

**Affiliations:** 1 Department of Psychology, Paris-Lodron-University of Salzburg, Salzburg, Austria; 2 Center for Cognitive Neuroscience, Paris-Lodron-University of Salzburg, Salzburg, Austria

**Keywords:** approach-avoidance task, implicit association task, food, external eating, touchscreen, reliability

## Abstract

Approach biases to foods may explain why food consumption often diverges from deliberate dietary intentions. Yet, the assessment of behavioural biases with the approach-avoidance tasks (AAT) is often unreliable and validity is partially unclear. The present study continues a series of studies that develop a task based on naturalistic approach and avoidance movements on a touchscreen (hand-AAT). In the hand-AAT, participants are instructed to respond based on the food/non-food distinction, thereby ensuring attention to the stimuli. Yet, this implies the use of instruction switches (i.e., ‘approach food – avoid objects’ to ‘avoid food – approach objects’), which introduce order effects. The present study increased the number of instruction switches to potentially minimize order effects, and re-examined reliability. We additionally included the implicit association task (IAT) and several self-reported eating behaviours to investigate the task’s validity. Results replicated the presence of reliable approach biases to foods irrespective of instruction order. Evidence for validity, however, was mixed: biases correlated positively with external eating, increase in food craving and aggregated image valence ratings but not with desire to eat ratings of the individual images considered within participants or the IAT. We conclude that the hand-AAT can reliably assess approach biases to foods that are relevant to self-reported eating patterns.

## Introduction

What and how much someone eats depends on explicit processes such as deliberate dietary intentions as well as on implicit responses to environmental food cues. While this cue reactivity expresses itself through cue-induced consumption and subjective food cravings, it is also evident in behavioural biases that motivate responses towards the respective food cue ( Boswell & Kober, 2015; Brockmeyer *et al*., 2015). These behavioural biases may play a pivotal role in the cascade of events that initiates and maintains dietary failure: approach bias towards food has been related to higher food intake when self-regulatory capacity is low, and it has been related to more uncontrolled eating in impulsive individuals ( Booth *et al*., 2018; Kakoschke *et al*., 2015; Kakoschke *et al*., 2017b). This does not seem to extend to individuals with clinically diagnosed binge eating however, who do not show an increased approach bias to food compared to healthy controls ( Paslakis *et al*., 2017). At the other end of the spectrum, a decrease or absence of approach bias towards food may help to explain the persistently reduced food intake in individuals with anorexia nervosa ( Neimeijer *et al*., 2019; Paslakis *et al*., 2016; for a review see Paslakis *et al*., 2020). Perhaps most importantly, a recent review has concluded that modification of approach bias can help reduce food consumption, thereby supporting a causal role for approach biases within normal and disordered food consumption ( Kakoschke *et al*., 2017a). Hence, research has begun to focus on the reliable assessment of behavioural biases so they can be measured and targeted for treatment in uncontrolled eaters.

Behavioural bias to food cues can be quantified using the approach-avoidance task (AAT). In the AAT, participants are required to approach and avoid stimuli from at least one category. An approach bias is inferred if a stimulus category, such as food, is approached faster than avoided, and this advantage for approach is larger than that of another stimulus category, such as office articles (e.g., Lender *et al*., 2018). It is necessary to compare two stimulus categories to control for stimulus-independent factors, such as differences in muscle strength or posture, which influence speed of approach and avoidance responses. Next to stimulus categories, the used representation of approach and avoidance (e.g. zooming stimuli, moving a manikin towards or away from stimuli, moving stimuli in 3D environment), the input devices (e.g. keyboards, computer mice, touchscreens, motion sensors) and the instruction types ( Krieglmeyer & Deutsch, 2010) affect psychometric properties of AAT implementations. Two different task instructions have been used. In the (more typical) irrelevant-feature AAT (e.g. Brockmeyer *et al*., 2015; Kakoschke *et al*., 2017a), participants must approach or avoid stimuli based on a feature of the stimulus that is unrelated to the bias being measured (e.g. approach stimuli with green frame and avoid those with a blue frame); in the relevant-feature AAT (e.g. Brignell *et al*., 2009; Lender *et al*., 2018; Meule *et al*., 2019), the participants must approach or avoid based on the stimulus category, thereby directing attention to the feature for which there might be an approach bias (e.g. approach food stimuli and avoid objects and vice versa). Feature-irrelevant AATs are appealing as they do not require an instruction switch halfway through the experiment, and are less susceptible to demand characteristics, thus allowing for stronger claims regarding *automatic* stimulus-response associations ( Chen & Bargh, 1999; Rinck & Becker, 2007). Yet, a meta-analysis including various stimulus categories (e.g. faces and affective words, Phaf *et al*., 2014) as well as direct task comparisons in the food domain suggest that only relevant-feature AATs have yielded significant and reliable approach biases, whereas biases in irrelevant-feature AATs were not significant or unreliable ( Lender *et al*., 2018; Meule *et al*., 2019; Phaf *et al*., 2014). Next to instruction, it matters whether it is the object or the participant that moves closer or further away. In set-ups using a joystick, approach bias effects are elicited primarily by the stimuli zooming in or out ( Krieglmeyer & Deutsch, 2010; Rinck & Becker, 2007) and accordingly, approach bias could be attained when stimuli were moved with simple key presses ( Becker *et al*., 2015; Peeters *et al*., 2012). However, it was shown that manipulating a stimulus’ position at a distance, as simulated with the zoom-feature, elicits smaller approach biases than moving oneself towards or away from a stimulus in a three-dimensional digital environment ( Rougier *et al*., 2018). Thus, unclear perspective and irrelevant-feature instructions may explain why only some studies find approach biases to foods or correlations with food craving and eating styles such as restrained and external eating ( Brignell *et al*., 2009; Brockmeyer *et al*., 2015; Lender *et al*., 2018; Maas *et al*., 2017a; Meule *et al*., 2019; Veenstra & de Jong, 2010) while others do not ( Matheson, 2018; Meule *et al*., 2020).

To improve the assessment of approach bias, we developed a new variant of the AAT. In this ‘hand-AAT’, participants slide their hand towards or away from a picture on a touchscreen, with movement direction depending on the stimulus category (relevant-feature: food vs. object). By moving one’s own, physical hand, there is no metaphoric ambiguity about whether the participant or the stimulus is moving, and participants perform actual ecologically valid approach and avoidance movements rather than producing abstract representations of approach and avoidance using artificial input devices such as joysticks or key presses. This task set-up previously yielded reliable approach biases to foods that correlated with explicit desire ratings in a healthy student population ( Kahveci *et al*., 2021), but unfortunately, the order of instruction blocks confounded inter-individual differences in approach bias: Both in the hand-AAT, as well as in a feature-relevant joystick AAT ( Kahveci *et al*., 2021; Wittekind *et al*., 2021), participants who avoid food and approach objects in the first block (inconsistent instructions) showed larger approach biases to food and stronger correlations with food craving than participants who approach food and avoid objects in the first block (consistent instructions). Additionally, two previous studies showed that the subjective desire to eat an individual food item is only correlated with reaction times on approach trials of that individual food item and not with reaction times on respective avoidance trials, making the value of avoid trials in the appetitive domain doubtful ( Kahveci *et al*., 2020; Kahveci *et al*, 2021). Given this, we speculate that block-order may confound bias size as participants gradually master the task and thus only respond in automatic fashion to approach-food trials when they occur in the second rather than the first block. Such block order effects were previously documented in the domain of implicit associations, and remedied through the introduction of multiple alternating blocks ( Messner & Vosgerau, 2010). In the current study, we thus chose to decrease the temporal primacy of one condition (instruction type, i.e., approach food, avoid objects) over another by presenting both conditions thrice and an alternating fashion rather than once.

As preregistered ( https://osf.io/ez7ka/), we expected that the hand-AAT would reliably detect a general approach bias to all foods in the task, based on the assumption that also low desired foods possess inherently rewarding properties thanks to their significance for human survival. As an indication of validity, we aimed to replicate the finding that more desired food stimuli are approached faster than less desired food stimuli ( Kahveci *et al*., 2021) and included the single category implicit association task (IAT) with approach and avoidance words to relate the AAT to another implicit measure that was validated for the assessment of motivational tendencies in the food context ( Kraus & Piqueras-Fiszman, 2016). Additionally, higher approach bias was hypothesized to relate to more self-reported food craving, as a marker of cue reactivity ( Brockmeyer *et al*., 2015). To further explore the relationship between bias scores and subjectively perceived cue-reactivity, we correlated the bias with changes in craving after exposure to foods cues in the hand-AAT, external eating, as well as overall liking and desire to eat ratings across food stimuli ( Brignell *et al*., 2009; Brockmeyer *et al*., 2015; Lender *et al*., 2018). To scope validity of this paradigm further, we assessed the relationships between AAT and IAT bias on the one hand and restrained eating and body mass index (BMI) on the other hand, as previous studies have argued that dietary lapses in individuals with obesity and in individuals restraining their food intake may be explained by strong implicit approach responses to food cues ( Kemps & Tiggemann, 2015; Veenstra & de Jong, 2010).

## Methods

### Participants

We recruited 59 students (24 male) of the University of Salzburg with announcements during lectures and with flyers on social media platforms. Three participants dropped out after the online questionnaire. As preregistered, 10 participants were excluded because they had an average desire-to-eat rating below 30 or above 70 and three more were excluded due to an excessive outlier or error rate on the AAT (>15%). Our final sample included 43 participants (17 male), aged between 18 and 30 years (mean [ *M*] = 22.95, standard deviation [ *SD*] = 3.54), and with a BMI between 18.02 and 39.67 kg/m² ( *M* = 23.13, *SD* = 4.54). We included the health and natural concern subscales of the eating motivation survey (TEMS) ( Renner *et al*., 2012) as we found stronger approach bias to low than to high caloric foods in a previous study in a similar student population and thus suspected that our participants may pay more attention to healthy eating than the general population. However, participants’ orientation towards healthy ( *M* = 4.59, *SD* = 1.04) and natural ( *M* = 4.06, *SD* = 1.31) foods did not differ from the health (Welch’s *t* (45) = 0.74, *p* = .462) and natural orientation (Welch’s *t* (45) = .54, *p* = .593) of the population.

### Questionnaires

Reliability values are based on the full sample. As Cronbach’s α systematically underestimates reliability, we additionally report McDonald’s ω ( Mcdonald, 1978; Revelle & Zinbarg, 2009; Sijtsma, 2009).

***TEMS – natural concern and health motivation.*** TEMS ( Renner *et al*., 2012) was used to compare this sample’s orientation towards natural and healthy food, compared to the general population. Each scale consisted of 5 statements about one’s reasons to eat food, rated on a 7-point likert scale. Reliability was good for the natural concern (α = .92, ω = .92) and health motivation subscale (α = .86, ω = .87).

***Food craving questionnaire – state (FCQ-S) and trait (FCQ-T-r).*** The German versions of the FCQ-S and FCQ-T-r ( Meule *et al*., 2014; Meule *et al*., 2012a) were used to measure state and trait food craving, respectively. Both scales consisted of 15 statements rated on a 5-point likert scale. Both scales had excellent reliability in this study (α = .90, ω = .90).

***Dutch eating behavior questionnaire (DEBQ).*** The three subscales of the DEBQ ( Van Strien *et al*., 1986) were used to measure emotional eating, external eating, and restrained eating. Each subscale consisted of 10 statements rated on a 5-point likert scale. All three subscales were reliable (emotional eating: α = .92, ω = .92; external eating: α = .87, ω = .86; restrained eating: α = .86, ω = .85).

***Other scales.*** The Perceived Self-Regulatory Success in Dieting scale ( Meule *et al*., 2012b) and the Positive And Negative Affect Schedule ( Watson *et al*., 1988) were administered but not preregistered/analysed.

### Materials and apparatus

The AAT was administered using a 23-inch iiyama ProLite T2336MSC-B2 touchscreen monitor with a resolution of 1920 × 1080 pixels, placed in portrait-format with a 10% tilt towards the participant.

The AAT included 24 object and 24 food images, selected from the food-pics_extended database ( Blechert *et al*., 2019) and the FRIDa database ( Foroni *et al*., 2013). As objects are used to control for stimulus-independent factors, they should not elicit behavioural response tendencies and we thus selected emotionally neutral objects based on normative ratings from the respective databases. The food images were drawn semi-randomly for each participant from a larger pool of 60 individually rated food items ^1^ to ensure an equal number of desired and non-desired foods. The stimuli’s variability on desire to eat was increased to examine trial-level correlations between individual RTs and item-specific desire to eat ratings. For the IAT, we selected the 12 most highly desired stimuli of the personalized stimulus set used in the AAT based on median split. Here we aimed for a more homogeneous stimulus set to achieve more precise IAT-biases for correlations on a participant-level.

**Figure 1.**
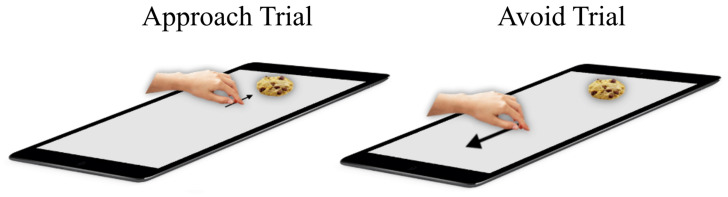
Hand-AAT. Description see text.

***AAT.*** In a typical AAT trial, participants placed their hand on a symbol centrally on the screen, and after a random delay between 300ms and 700ms, a stimulus was displayed on the distal side of the touchscreen. Participants approached or avoided the stimulus by sliding their hand towards it or away from it, respectively ( Figure 1). After approaching a stimulus, it ‘snapped’ to the hand and was moved back to the center of the screen along with the hand. Stimuli were avoided by moving the hand away from the stimulus and towards an avoidance zone at the proximal side of the touchscreen. After avoiding a stimulus, the stimulus disappeared. Participants completed a 12-trial practice block, followed by six blocks with 48 trials each. At the start of each block, participants were instructed to either approach foods and avoid objects (consistent blocks), or to avoid foods and approach objects (inconsistent blocks). This alternated from one block to the next and the order was counterbalanced between participants. Stimuli were shown in semi-random order to ensure each stimulus category was not repeated more than thrice ( Wiers *et al*., 2010). An error was recorded if participants lifted their hand or initiated a movement in the wrong direction. The time from stimulus onset until movement onset was chosen as the reaction time (RT) measure.

On approach trials, the participant slides their hand from the middle towards the food/object and on avoid trials the participants slides their hand from the middle in the direction opposite to the food/object stimulus.

***Single-category implicit association task (IAT).*** During the IAT, participants sorted 6 German approach words (e.g., ‘approach’, ‘grab’), 6 avoidance words ^2^ (e.g., ‘avoid’, ‘remove’), and 12 food images into categories displayed at the left or the right side of the screen using the E and I keys, respectively. “Approach” was always displayed at one side, and “Avoidance” at the other; this was counterbalanced by the participant. In the congruent block, participants had to sort food images to the same side as the approach words, and in the incongruent block, participants had to sort the food images to the same side as the avoid words. These congruent and incongruent blocks were administered after a practice block, and the order in which they were presented was counterbalanced by participant in accordance with the AAT, such that participants received the same block order in both tasks, to facilitate the detection of any correlations between them.

The practice block consisted of 24 trials in which participants sorted approach/avoidance related words. The subsequent congruent and incongruent blocks featured these words as well as food images. These latter blocks consisted of 84 trials, of which 24 were food trials, 24 were words to be categorized to the same side as the food images, and 36 were words to be categorized to the other side. This unequal division was required to be able to balance the number of responses on either side, while having two stimulus categories on one side and one on the other side ( Karpinski & Steinman, 2006).

### Procedure

**Figure 2.**
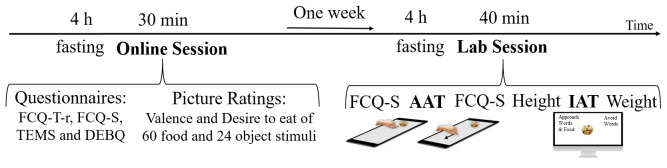
Procedure.

The study was conducted with permission granted by the ethics committee of the Paris-Lodron University of Salzburg (EK-GZ: 27/2018), in accordance with the Declaration of Helsinki and participants provided written consent to study procedures (displayed in Figure 2). Prior to the start of the study, participants were instructed to fast for at least four hours, with the intent of increasing their food cravings. After these four hours, they completed online-versions of the FCQ-T-r, FCQ-S, TEMS and DEBQ, and rated all food and object stimuli on valence, and all food stimuli on desire-to-eat. Exactly one week after this online-session, participants fasted again for at least four hours and were then invited to the lab. Here they completed the FCQ-S, followed by the AAT, and the FCQ-S again, afterwards their height was measured, the IAT was administered, their weight was measured, and they were reimbursed after signing a form of consent. The one week between the online and the lab session was intended to wash out an **y** effects of the stimulus rating and to allow time for customizing the lab-tasks with regard to individual stimulus sets. 

### Data processing

Data were pre-processed and analyzed as pre-registered (see attached R scripts). First, RTs were excluded if they were above 1500ms or below 200ms, or if the response was incorrect; then, RTs were square-root transformed to improve normality; after this, RTs were excluded if they deviated more than 3 *SD*s from the participant’s mean.

For the multilevel analyses, we included all level 1 fixed effects also as random effects nested under stimulus, and we further included random intercepts per stimulus and random slopes for trial number per block per subject. Significance of highest-order model terms was tested by comparing a model with the effect to a model without the effect using a Wald chi-square test. The reported standardized regression coefficients are based on the full model.

We computed bias scores for the AAT and IAT in accordance with the D-score algorithm outlined by Greenwald *et al*., 2003 as this algorithm was shown to produce the most reliable and externally valid IAT bias scores. In accordance with this algorithm, RTs below 10s were included and error trials were replaced by the correct block mean plus a 600ms penalty. D-scores were computed by subtracting the mean RT for each consistent block from the mean RT of the adjacent inconsistent block, dividing the result by the standard deviation of the RTs in those blocks, and averaging the D-scores of all sets of two blocks to result in a final D-score. Food-approach associations are represented by the IAT D-score, and food approach biases are represented the AAT D-score. For both, higher scores represent stronger biases.

## Results

### Reliability

Bootstrapped split-half reliability coefficients were computed using the AATtools package ( Kahveci, 2020) for R ( R Core Team, 2019). The sample was split randomly, outliers were excluded, and bias scores were computed in accordance with the Methods section, and scores from both halves were correlated. This process was repeated 10000 times and the resulting split-half correlations were averaged and corrected for halved test length. The AAT was reasonably reliable for an implicit measure, *r*_SB_ = .64, as was the IAT, *r*_SB_ = .66.

### Bias

**Figure 3.**
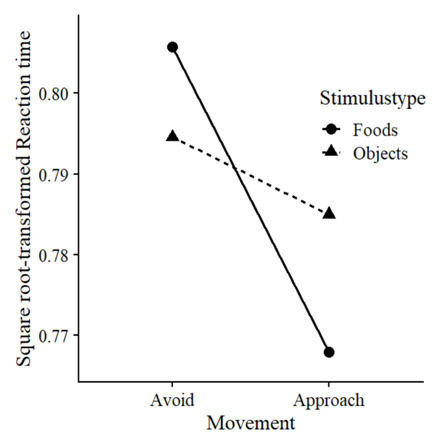
Behavioural approach bias to foods.

We examined whether there was a greater behavioural approach bias for foods compared to objects. We predicted square root-transformed RTs using fixed and random factors for Movement (0 = avoid, 1 = approach) and Stimulustype (0 = object, 1 = food), as well as with random intercepts per stimulus and random slopes of trial number per block per participant, as described in Equation 1. Indicative of a bias, Movement and Stimulustype interacted, χ² (1) = 21.20, *p* < .001, *β* = -.128. Follow-up analyses confirmed that, compared to objects, foods were avoided slower, χ² (1) = 6.63, *p* = .010, *β* = .057, ΔRT = 16ms, and approached faster, χ² (1) = 18.00, *p* < .001, *β* = -.095, ΔRT = 28ms ( Figure 3).

Mean reaction times in seconds per condition.

- sqrtRT ~ Movement * Stimulustype + (Movement * Stimulustype | Subject) + (1 | Stimulus) + (TrialNumber – 1 | Subject/Block)                        (1)

As for the IAT, D-scores significantly differed from zero, indicating an association between food and approach, *t* (42) = 3.00, *p* = .003. There was no significant relationship between behavioural approach bias for highly desired stimuli and implicit associations for highly desired stimuli, *r* (41) = -.12, *p* = .446. Note, both biases were calculated using the same stimuli. This was possible as stimuli displayed in the IAT were also displayed on half of the AAT-trials.

### Desire to eat

To investigate the effect of the participant’s desire to eat specific foods on behavioural approach bias, we predicted square root-transformed RTs with movement, desire, and their interaction, as fixed and random effects, as well as random intercepts per stimulus and random slopes but no intercepts for trial number per block, as depicted in Equation 2. There was no larger difference between approach and avoidance reaction times for stimuli that were more desired, χ ^2^ (1) = .87, *p* = .350, *β* = .028.

- sqrtRT ~ Movement * Desire + (Movement * Desire | Subject) + (1 | Stimulus) + (TrialNumber – 1 | Subject/Block)                                          (2)

### Craving, BMI, and eating behaviour

**Figure 4.**
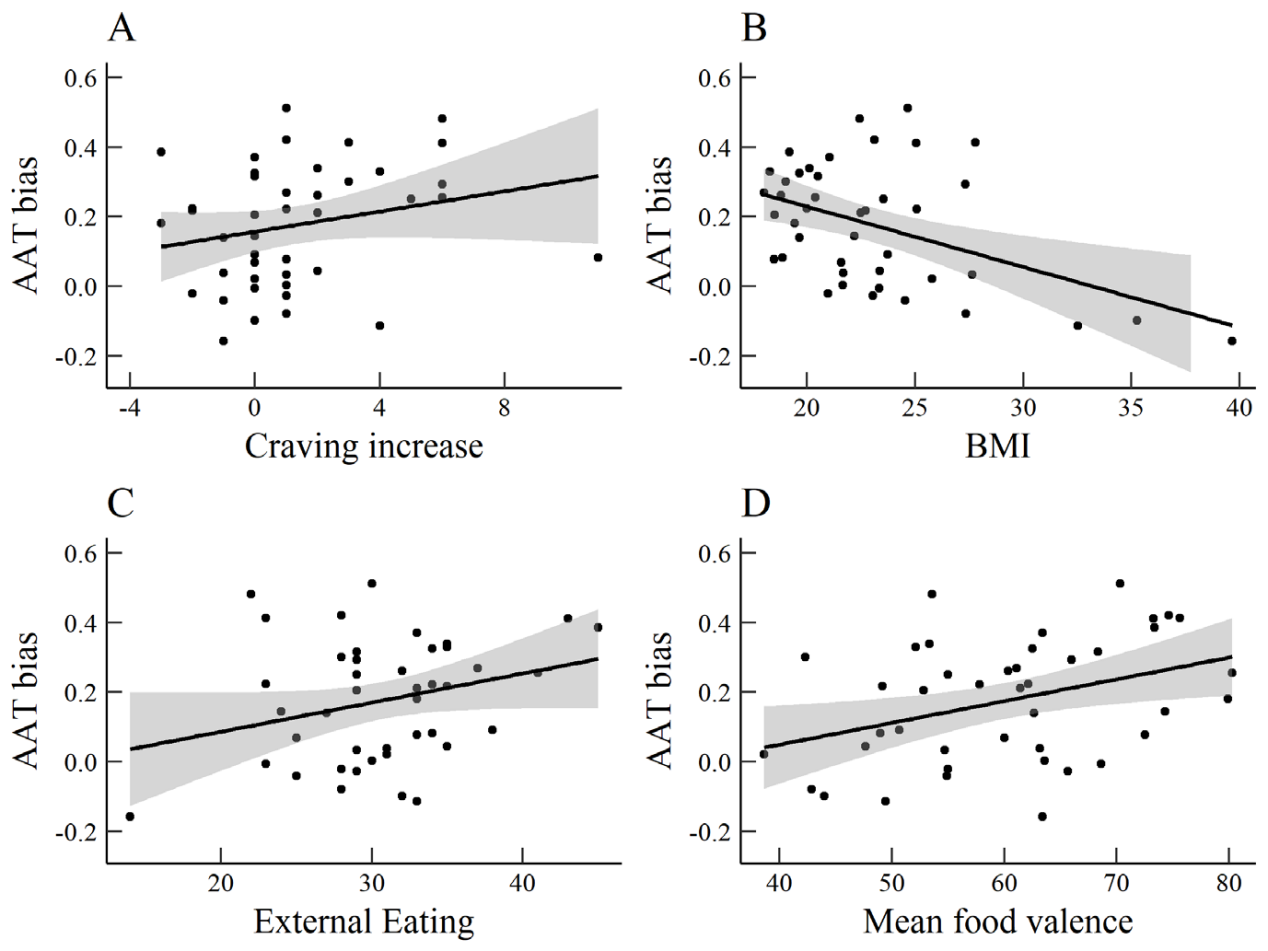
Scatterplots of significant correlations between AAT bias and **a**) increase of the FCQ-S from pre to post-AAT, **b**) body mass index, **c**) DEBQ-external eating and **d**) mean food valence.

We explored correlations between AAT and IAT D-scores on the one hand, and the DEBQ subscales, state and trait food craving, BMI, and mean ratings of the foods on the other hand. Correlations are listed in Table 1. Higher external eating scores related to higher AAT approach bias and IAT association bias scores. AAT bias correlated positively with the increase in craving from pre-test to post-test and with mean ratings of food valence, but negatively with BMI ( Figure 4). The latter effect must be interpreted cautiously, as only three participants with obesity (BMI > 30) were included in the sample, and the correlation was non-significant ( *r* (38) = -.22, *p* = .177) after those participants were excluded. It should also be noted that power to detect a medium correlation ( *r* = .3) was suboptimal (1 - β = .51), which may have obscured true effects while moving spurious effects to the foreground.

**Table 1.** Correlations between AAT and IAT D-Scores on the one hand and self-reports on the other hand. *significance at trend-level (α < .1).

	AAT bias		IAT bias	
	*r* (41)	*p*	*r* (41)	*p*
Pre-AAT craving (FCQ-S)	.05	.733	.16	.292
Post-AAT / Pre-IAT craving (FCQ-S)	.23	.143	.13	.401
Δ Craving increase (FCQ-S)	**.31**	**.040**	-.03	.858
Trait craving (FCQ-T-r)	.02	.894	.13	.389
Body Mass Index	**-.45**	**.002**	.19	.225
External eating (DEBQ)	**.37**	**.016**	**.32**	**.036**
Restrained eating (DEBQ)	-.06	.684	.29 *	.057
Mean food valence	**.38**	**.012**	-.28 *	.074
Mean food desire	.30 *	.052	.02	.900

### Block order effects

We explored whether the demonstrated behavioural approach bias of the AAT was affected by whether participants received the consistent or inconsistent block first. We predicted square root-transformed RTs using fixed and random effects for Movement, Stimulustype, and their interactions, fixed effects for Order and its interactions with the other fixed effects, as well as random intercepts per stimulus and random slopes but no intercepts for trial number per block, as described in Equation 3. Block order did neither affect the approach bias toward foods when analysing six blocks, χ ^2^ (1) = 3.30, *p* = .069, *β* = .063 nor when analysing the first four, χ ^2^ (1) = 1.58, *p* = .21, *β* = .067, or the first two blocks χ ^2^ (1) = 0.53, *p* = .47, *β* = .055.

- sqrtRT ~ Movement * Stimulustype * Order + (Movement * Stimulustype | Subject) + (1 | Stimulus) + (TrialNumber-1 | Subject/Block)          (3)

Accordingly, D-scores ( *M* = .17, *SD* = .16) for participants starting with the inconsistent block did not differ significantly from D-scores ( *M* = .16, *SD* = .22) for participants starting with the consistent block (Welch’s *t* (32) = .3, *p* = .800). As for the IAT, approach associations did not differ significantly for block order (Welch’s *t* (40) = 1, *p* = .200).

## Discussion

We found that participants had a behavioural approach bias toward food in the AAT and an implicit association between food and approach in the IAT. While being unrelated to each other, both biases were stronger in individuals with higher external eating. Larger AAT biases were further found in participants reporting increases in food craving over the course of the experiment. We thus demonstrated a relationship between forms of cue-reactivity as expressed through implicit approach responses, cue-induced craving, and eating style. Yet, individually more desired food items were not approached faster, even though larger AAT biases were related to higher mean food liking, and neither the AAT biases nor the IAT biases correlated with state craving, trait craving, restrained eating, or general desire to eat different foods.

The lack of an association between AAT and IAT scores is not an uncommon finding in the eating literature ( Maas *et al*., 2017a; Woud *et al*., 2016) and in implicit bias research more broadly ( Pieters *et al*., 2014), with some researchers even finding a negative correlation between the two ( Larsen *et al*., 2014; Warschburger *et al*., 2018). These findings suggest that the AAT measures the association between stimuli and directional movements, whereas the approach-avoidance IAT measures the association between stimuli and words. The associations with approach-avoidance words do not necessarily overlap with actual behavioural tendencies and can directly oppose each other, for example in dieters who have an approach bias towards food but associate it with avoidance-related self-talk ( Wiers *et al*., 2017), hence supporting a more ‘verbal’ nature of the IAT, at least relative to the AAT. Despite being unrelated to each other in the current study, both tasks were associated with external eating, the tendency to eat in response to external cues rather than internal ones such as hunger. This suggests that some participants may display external eating due to strong cue-elicited approach responses, while others may display external eating due to a more cognitive association between food and consumption, for example due to food-related beliefs and cultural norms.

We could not replicate the finding that interpersonal differences in the desire to eat individual food items predict approach bias for those individual food items ( Kahveci *et al*., 2020; Kahveci *et al*., 2021). This may be because this study featured a one-week delay between the desire to eat ratings and approach bias measurement, whereas the aforementioned studies collected ratings directly after measurement of approach bias. The relationship between approach bias and food preferences may thus be momentary, as the desire for specific foods changes within days ( Reichenberger *et al*., 2018) and the relationship between implicit associations and consumption behaviour was only found under high craving and hunger ( Richard *et al*., 2019). While preferences for specific food items change over time, the general tendency to either like or dislike most food types may be more stable (e.g. picky eating; Kauer *et al*., 2015). In line with this, we showed that higher mean food liking relates to stronger approach bias despite the one-week delay. On the most general level, AAT correlates come both from state-domain (e.g. desire for specific foods at that moment) and trait-domain measures (e.g. external eating, general food liking). Future research should thus invest into decomposing validity effect into proximal states and their role in mediating more distal traits.

One important methodological step is that the present task successfully remedied the confounding effect of block order on approach bias scores, which was found in the previous feature-relevant AATs ( Kahveci *et al*., 2021; Wittekind *et al*., 2021). Such block order effects introduce differences in participants’ bias scores which are unrelated to the participant’s inherent approach bias, and thus reduce the correlations between the measured bias and external measures. As instruction switches are inevitable in feature-relevant AATs, the removal of block-order effects is pivotal for the further development of this ‘strain’ of AAT methods. In the current study, biases were found regardless of whether the inconsistent or consistent block started the block sequence. Yet, also the bias during the first two or four blocks was unaffected by instruction order and thus, increasing the number of blocks to six was not the only reason block order effect disappeared. Possibly, the current task was easier to master than the previous one, which reduced the influence of learning effects that would otherwise slow reaction times during whichever block is presented first. We assume that the current task was easier to master because stimuli were displayed only in one position on the screen, whereas the previous study changed the location of the stimuli halfway through each block. Therefore, we recommend an extended practise phase to decrease the artificial differences in behavioural approach bias when it is not feasible to avoid counterbalancing block order.

Reliability of the hand-AAT may seem promising when considering the ‘reliability crisis’ in the broader field of cognitive bias measurement ( LeBel & Paunonen, 2011; McNally, 2019) and was in line with that of another feature-relevant AAT in the food domain ( Kahveci *et al*., 2020). However, two other feature-relevant AATs using either a joystick or symbolic manikin to approach and avoid stimuli unrelated to food attained higher reliability ( Krieglmeyer & Deutsch, 2010), just like our previous version of the hand-AAT ( Kahveci *et al*., 2021). Reliability of this previous AAT may have been numerical higher because the study featured stimuli with a more narrow range of stimulus valence, and because the strong block-order effects likely increased the variability of approach bias scores. The reliability and power of the current paradigm may be improved with more trials and with stimulus sets standardized on graspability ( Baker *et al*., 2020). 

The current study is part of a line of research geared towards improving the assessment of food-related approach biases through the use of manual responses on a touchscreen. Block-order effects that were found in previous studies were remediated, and thus a strong confound of bias size was eliminated in the current task set-up. Accordingly, the bias scores attained concurrent validity: larger approach bias related to stronger cue-induced craving, external eating and food liking. We further found that food approach bias was unrelated to food approach associations, despite the fact that both measures related to external eating; this finding may inform future intervention studies, as it suggests that some people may benefit from approach bias modification and others from the modification of implicit associations. As the task showed first signs of validity, no block order confounds, and attained a reliability slightly under what is considered sufficient (r = .7; Rammstedt, 2004), it may be a good starting point for future research in the measurement and modification of automatic appetitive responses.

## Data availability

OSF: Improving the touchscreen-based food approach-avoidance task: remediated block-order effects and initial findings regarding validity

https://doi.org/10.17605/OSF.IO/EZ7KA ( van Alebeek *et al*., 2021)

This project contains the following underlying data:

- Raw data files:
- Anthropometry.sav (height and weight measured during the lab session)
- 1_SCIAT.csv – 61_SCIAT.csv (separate IAT files for each participant)
- 1_2019-11-07-17-25.csv - 61_2020-02-04-16-16.csv (separate AAT files for each participant)
- home.sav (demographics, FCQ-T-r, the TEMS, perceived self-regulatory success in dieting scale, DEBQ, FCQ-S and individual stimulus rating on valence and desire to eat assessed during the online survey)
- post.sav (the FCQ-S administered subsequent to the AAT)
- pre.sav (the positive and negative affect schedule and the FCQ-S administered prior to the AAT)
- Pre-processed data files:
- HandSRT2_IAT_longformart.csv (trial-level IAT data for all participants)
- HandSRT2_preppeddata.csv (trial-level AAT data for all participants)
- HandSRT2_masterfile.csv (participant-level questionnaire sum scores and aggregated AAT as well as IAT scores)
- Analyses and pre-processing scripts:
- IATextraction.R (R-code to merge and pre-process IAT data as well as to compute IAT D-scores)
- Datapreparation.R (R-code to merge and pre-process AAT and questionnaire data and to subsequently combine them with the pre-processed IAT data)
- Analyses.R (R-code used for analyses of results)

Data are available under the terms of the Creative Commons Zero "No rights reserved" data waiver (CC0 1.0 Public domain dedication).
