# Supplementary material for: Improving the touchscreen-based food approach-avoidance task: remediated block-order effects and initial findings regarding validity
Source: Open Res Eur. 2021 Aug 20;1:15. Originally published 2021 Mar 24. [Version 3] doi: 10.12688/openreseurope.13241.3 (PMC10445824; doi:10.12688/openreseurope.13241.3)
Supplement: Peer review history

# Peer review: Reviewer response for version 3

**DOI:** 10.21956/openreseurope.15166.r27668

**Published:** 2021-10-12

**Authors:** Philipp Schroeder

**Affiliations:** 1 Department of Psychology, Clinical Psychology and Psychotherapy, University of Tübingen, Tübingen, Germany

The research article by van Alebeek *et al*. reports a single experiment with healthy volunteers who completed behavioral assessment using a mobile approach-avoidance task (AAT) with task-relevant instructions together with an implicit association test (IAT) and several self-report measures of food craving and eating behavior. The goal of the research was to investigate whether faster approach to food in the AAT was dependent on block-order in a block design with increased number of instruction switches. The study furthermore investigates the validity of the results gathered from the AAT and reports mixed results: The authors observed some associations with self-reported eating behavior and there was no association with the IAT, suggesting discriminant validity. Individual desire ratings of the food stimuli were collected a week before behavioral testing but did not predict approach or avoid reaction times. Furthermore, the authors argue that including more switches between the task-relevant classification dimensions mitigated a previously observed effect of block order.

 Overall, the motivation and results from the study are well described and the manuscript has apparently been improved from the previous review cycles. Limitations of the study, particularly the very low sample size for correlational analyses, are acknowledged and the manuscript is very clear. Although their statistical approach to interpret a null effect could be more convincing (e.g., by using Bayesian analysis or equivalence test, or comparing the frequent-switch version of this submission with one of their previous data sets), their conclusion of a mitigated block order effect seems appropriate. I do not have additional comments and approve this version for indexing.

If applicable, is the statistical analysis and its interpretation appropriate?

Yes

Is the study design appropriate and is the work technically sound?

Yes

Is the work clearly and accurately presented and does it engage with the current literature?

Yes

Are the conclusions drawn adequately supported by the results?

Yes

Are sufficient details of methods and analysis provided to allow replication by others?

Yes

Are all the source data and materials underlying the results available?

Yes

Reviewer Expertise:

psychology

I confirm that I have read this submission and believe that I have an appropriate level of expertise to confirm that it is of an acceptable scientific standard.

# Response

**Published:** 2021-10-12

**Authors:** Hannah van Alebeek

**Affiliations:** University of Salzburg, Austria

Dear Philipp Schröder, thanks a lot for reviewing our manuscript and the changes we made in response to the previous comments. Kind regards, Hannah van Alebeek

# Peer review: Reviewer response for version 3

**DOI:** 10.21956/openreseurope.15166.r27452

**Published:** 2021-10-05

**Authors:** Naomi Kakoschke

**Affiliations:** 1 Health and Biosecurity, the Commonwealth Scientific and Industrial Research Organisation (CSIRO), Adelaide, Australia; 2 Turner Institute for Brain and Mental Health and School of Psychological Sciences, Monash University, Melbourne, Australia

I am happy with the revisions. I approve this article for indexing.

If applicable, is the statistical analysis and its interpretation appropriate?

Yes

Is the study design appropriate and is the work technically sound?

Yes

Is the work clearly and accurately presented and does it engage with the current literature?

Yes

Are the conclusions drawn adequately supported by the results?

Yes

Are sufficient details of methods and analysis provided to allow replication by others?

Yes

Are all the source data and materials underlying the results available?

Yes

Reviewer Expertise:

NA

I confirm that I have read this submission and believe that I have an appropriate level of expertise to confirm that it is of an acceptable scientific standard.

# Peer review: Reviewer response for version 2

**DOI:** 10.21956/openreseurope.14737.r27041

**Published:** 2021-06-15

**Authors:** Naomi Kakoschke, Rajeshwari Muthukumaran

**Affiliations:** 1 Health and Biosecurity, the Commonwealth Scientific and Industrial Research Organisation (CSIRO), Adelaide, Australia; 2 Turner Institute for Brain and Mental Health and School of Psychological Sciences, Monash University, Melbourne, Australia; 3 Cognitive and Behavioural Neuroscience Laboratory, Department of Humanities and Social Sciences, Indian Institute of Technology Bombay, Mumbai, Maharashtra, India

We thank the authors for their work in revising the paper. We would like to request one minor change, namely, that the authors add a brief concluding paragraph to the end of the discussion section. We would then be happy to accept the manuscript for indexing based on the most recent revisions.

If applicable, is the statistical analysis and its interpretation appropriate?

Yes

Is the study design appropriate and is the work technically sound?

Yes

Is the work clearly and accurately presented and does it engage with the current literature?

Yes

Are the conclusions drawn adequately supported by the results?

Yes

Are sufficient details of methods and analysis provided to allow replication by others?

Yes

Are all the source data and materials underlying the results available?

Yes

Reviewer Expertise:

Cognitive biases, implicit processes, food cues, eating behaviour, approach-avoidance assessment and modification.

We confirm that we have read this submission and believe that we have an appropriate level of expertise to confirm that it is of an acceptable scientific standard, however we have significant reservations, as outlined above.

# Response

**Published:** 2021-08-20

**Authors:** Hannah van Alebeek

**Affiliations:** University of Salzburg, Austria

Dear Dr. Kakoschke and Ms. Muthukumaran,

 On behalf of my co-authors and myself, I thank you again for your helpful remarks and for making the effort to evaluate how we implemented them. As you suggested, we added a concluding paragraph to summarize the central points of this work. In case of further questions or comments, please feel free to contact us any time.

 Kind regards, Hannah van Alebeek

# Peer review: Reviewer response for version 1

**DOI:** 10.21956/openreseurope.14311.r26590

**Published:** 2021-04-09

**Authors:** Naomi Kakoschke, Rajeshwari Muthukumaran

**Affiliations:** 1 Cognitive and Behavioural Neuroscience Laboratory, Department of Humanities and Social Sciences, Indian Institute of Technology Bombay, Mumbai, Maharashtra, India; 2 Health and Biosecurity, the Commonwealth Scientific and Industrial Research Organisation (CSIRO), Adelaide, Australia; 3 Turner Institute for Brain and Mental Health and School of Psychological Sciences, Monash University, Melbourne, Australia

Implicit learning underlies approach-avoidance tendencies and related food habits. This study is an extension of a previous AAT model and is aimed at improving its reliability by reducing block order effects. Overall, the document was well written and comprehensive for a single experiment paper. The task description for hand-AAT is well described and the figure aids the interpretation of the novel assessment methodology. However, there are some minor issues that need to be addressed before the manuscript can be considered suitable for indexing.

**Introduction**

 1.    This study is built upon the yet to be published previous study. Therefore, a brief overview of the findings from the previous study could be included to describe what were the order effects were, how did it affect the approach biases. As it currently stands, this information is scattered throughout the document. 

 2.     The second paragraph starts with explaining AAT and moves to introduce why some studies have demonstrated an approach bias while others have not. Inclusion of a sentence to introduce/summarize that these differences are discussed could help with the readability.

 3.    The authors should note that approach biases involve comparing approach relative to avoidance movements for a single category (e.g. unhealthy food) of stimuli as only one picture type is presented at a time on the screen. A comparison of approach bias scores across categories (e.g., approach for healthy versus unhealthy food) is possible, but not a required component. In addition, more than two categories can be included, e.g., unhealthy food, healthy food, and neutral cues.

 4.    It would also be useful to mention the reason that some researchers have used an irrelevant feature version of the AAT (i.e., to assess implicit processes) rather than a relevant feature version.

 5.    It would be helpful to provide a rationale for modifying the motor response component of the AAT instead of modifying other aspects such as the instructions (e.g., is it more ecologically valid to use hand movements than a joystick or key press?)

**Methods**

 7.    The authors should consider if such a detailed description is necessary or if they could simply mention that 3 participants were not included in the final sample?

 “Subsequent to the online-questionnaires, two participants cancelled their appointment and one did not show up to the lab-session.”

 8.    More information is needed about the TEMS, e.g., the number of items, what is it designed to measure and why only two subscales were included rather than the full scale.

 9.    In the task description of IAT, the location in which the words 'approach’, ‘avoidance’ and ‘food’ appear on the screen could be clearer. However, while reading the next paragraph it becomes evident that the approach side will always be the food side. I would request the authors to consider if this can be explained better.

 10.    Sorry if I missed it, but can the authors clarify the purpose of the two sessions and what differed between them? Perhaps it would also be helpful to provide a study design statement.

 11.    The authors should provide a rationale for why there is a 600ms penalty when scoring the IAT, “For the computation of AAT and IAT D-scores, all RTs below 10s were included and error trials were replaced by the correct block mean plus a 600ms penalty.”

**Results**

 12.    Was the relationship between approach bias and implicit associations also estimated for less desired stimuli? On a related note, how were highly desired versus less desired stimuli defined?

**Discussion**

 13.    The term cue-reactivity is first introduced in discussion; it might be worthwhile to use and define this term in the introduction itself.

 14.    In the first paragraph, there is a brief summary in the format of result 1, result 2…etc. The subsequent discussion can follow the same order of results as presented in the brief summary to improve the readability. For example, in the second paragraph, the explanation starts with result n (lack of correlation between IAT and AAT being the last) and then connects it with result 1.

 15.    Differentiation between AAT and IAT has been discussed in terms of behavioral and cognitive aspects of approach and avoidance. The last statement mentions these constructs might not overlap. It would be useful to add some references to support the statement here. Further, the study has attempted to mention the following ‘despite’ the lack of correlation between IAT and AAT.

 “These findings underline that the two tasks measure different concepts: the AAT measures the readiness to perform approach and avoidance movements in response to a stimulus, while the approach-avoidance IAT measures associations between the stimulus and the cognitive concepts of approach and avoidance – associations that do not necessarily overlap with actual behavioural tendencies.”

 16.    The relationship between BMI and AAT/IAT has been discussed even though only 3 participants had a BMI>30. Since the study finding is not in line with previous findings; other than the 3 participants explanation, there is not much to explain the current finding. The authors could reconsider inclusion of this paragraph? (purely because it does not add any more value to the paper).

 17.    The explanation for the ‘interpersonal differences in the desire to eat individual food items predict approach bias for those individual food items’ appears to be well written as it has well connected with the momentary craving aspect. The effect of valence of food had been correlated with AAT. The discussion does not seem to explain this effect.

 18.    In addition, “it is not uncommon that feature-relevant AATs attain reliability estimates in the upper range across implicit measures (Gawronski et al., 2011) and reliability in current task set-up was lower than in our previous version of the hand-AAT (Kahveci et al., submitted)”. It might be relevant to mention if the reliability was in line with the previous feature-relevant AATs as that might be one of the positive aspects of the study as well.

If applicable, is the statistical analysis and its interpretation appropriate?

Yes

Is the study design appropriate and is the work technically sound?

Yes

Is the work clearly and accurately presented and does it engage with the current literature?

Yes

Are the conclusions drawn adequately supported by the results?

Yes

Are sufficient details of methods and analysis provided to allow replication by others?

Yes

Are all the source data and materials underlying the results available?

Yes

Reviewer Expertise:

Cognitive biases, implicit processes, food cues, eating behaviour, approach-avoidance assessment and modification.

We confirm that we have read this submission and believe that we have an appropriate level of expertise to confirm that it is of an acceptable scientific standard, however we have significant reservations, as outlined above.

# Response

**Published:** 2021-06-04

**Authors:** Hannah van Alebeek

**Affiliations:** University of Salzburg, Austria

Dear Dr. Kakoschke and Ms. Muthukumaran,

 My co-authors and I are very thankful for having the opportunity to improve our paper by carefully considering and implementing your insightful comments. We apologize that this response is posted after you uploaded your second review and thus, was not able to facilitate your second review process. However, it is our pleasure to outline in a one-on-one fashion how we addressed your comments, which may help you and other readers to orient through the second version of the paper.

**Comment 1**: *This study is built upon the yet to be published previous study. Therefore, a brief overview of the findings from the previous study could be included to describe what were the order effects were, how did it affect the approach biases. As it currently stands, this information is scattered throughout the document.*

**Response:** We are happy to announce that this paper has been published and is now openly assessable. To improve readability, the main findings are summarized in paragraph three.

**Comment 2:** *The second paragraph starts with explaining AAT and moves to introduce why some studies have demonstrated an approach bias while others have not. Inclusion of a sentence to introduce/summarize that these differences are discussed could help with the readability.*

**Response:** Based on your suggestion, we included an introductory sentence that gives examples of different AAT implementations in the second paragraph.

**Comment 3:** *The authors should note that approach biases involve comparing approach relative to avoidance movements for a single category (e.g. unhealthy food) of stimuli as only one picture type is presented at a time on the screen. A comparison of approach bias scores across categories (e.g., approach for healthy versus unhealthy food) is possible, but not a required component. In addition, more than two categories can be included, e.g., unhealthy food, healthy food, and neutral cues.*

**Response: **We agree that the AAT is used with differing number of stimulus categories and accordingly describe this more openly in the beginning of paragraph two. Yet, we added a sentence why we think it is important to compare responses between two stimulus categories.

**Comment 4:** *It would also be useful to mention the reason that some researchers have used an irrelevant feature version of the AAT (i.e., to assess implicit processes) rather than a relevant feature version.*

**Response:** According to your suggestion, we put the irrelevant and relevant-feature AAT in context to each other by summarizing the main advantages of feature-irrelevant AATs in paragraph two.

**Comment 5:** *It would be helpful to provide a rationale for modifying the motor response component of the AAT instead of modifying other aspects such as the instructions (e.g., is it more ecologically valid to use hand movements than a joystick or key press?)*

**Response:** We included a sentence in paragraph three on advantages of using one’s own hand instead of artificial input devices.

**Comment 7:** *The authors should consider if such a detailed description is necessary or if they could simply mention that 3 participants were not included in the final sample? “Subsequent to the online-questionnaires, two participants cancelled their appointment and one did not show up to the lab-session.”*

**Response:** To make the manuscript more concise, we decreased the detail of this sentence in the participant section.

**Comment 8:** *More information is needed about the TEMS, e.g., the number of items, what is it designed to measure and why only two subscales were included rather than the full scale.*

**Response:** We added additional information for all questionnaires and described the rational for including the TEMS in the participant section.

**Comment 9:** *In the task description of IAT, the location in which the words 'approach’, ‘avoidance’ and ‘food’ appear on the screen could be clearer. However, while reading the next paragraph it becomes evident that the approach side will always be the food side. I would request the authors to consider if this can be explained better.*

**Response:** We revised the description of the IAT and specified that food images either are sorted together with approach words or together with avoid words depending on the block type.

**Comment 10:** *Sorry if I missed it, but can the authors clarify the purpose of the two sessions and what differed between them? Perhaps it would also be helpful to provide a study design statement.*

**Response:** To enhance understanding of the studies procedures, we added a figure and describe why there was a one-week delay in the *procedure* section.

**Comment 11:** T *he authors should provide a rationale for why there is a 600ms penalty when scoring the IAT, “For the computation of AAT and IAT D-scores, all RTs below 10s were included and error trials were replaced by the correct block mean plus a 600ms penalty.”*

**Response:** In the Data processing section, we describe that we based pre-processing decisions on the study by Greenwald et al., 2003.

**Comment 12:** *Was the relationship between approach bias and implicit associations also estimated for less desired stimuli? On a related note, how were highly desired versus less desired stimuli defined?*

**Response:** As less desired stimuli were not included in the IAT, we are not able to correlate AAT and IAT bias for less desired stimuli. We outlined our reasons for including a larger range of desired stimuli in the AAT than in the IAT in the Materials and apparatus Section. Based on the pre-rating we selected high and low desired stimuli for each participant and separated both based on median split. On a related note, we specified in the introduction that we expect approach bias in the AAT across high and less desired stimuli as all edible foods can be regarded as positive stimuli.

**Comment 13**: *The term cue-reactivity is first introduced in discussion; it might be worthwhile to use and define this term in the introduction itself.*

**Response:** To remove this inconsistency, we describe how cue-reactivity can be indexed using self-reports and reaction time tasks in the first paragraph of the introduction.

**Comment 14:** *In the first paragraph, there is a brief summary in the format of result 1, result 2…etc. The subsequent discussion can follow the same order of results as presented in the brief summary to improve the readability. For example, in the second paragraph, the explanation starts with result n (lack of correlation between IAT and AAT being the last) and then connects it with result 1.*

**Response: **To enhance the structure of the paper, we rearranged the first paragraph of the discussion.

**Comment 15:** *Differentiation between AAT and IAT has been discussed in terms of behavioral and cognitive aspects of approach and avoidance. The last statement mentions these constructs might not overlap. It would be useful to add some references to support the statement here. Further, the study has attempted to mention the following ‘despite’ the lack of correlation between IAT and AAT.*

*“These findings underline that the two tasks measure different concepts: the AAT measures the readiness to perform approach and avoidance movements in response to a stimulus, while the approach-avoidance IAT measures associations between the stimulus and the cognitive concepts of approach and avoidance – associations that do not necessarily overlap with actual behavioural tendencies.”*

**Response:** The assumption that the tasks outcome measures do not overlap is driven by the negative or insignificant correlations despite using the same stimuli and preprocessing routine. Yet, we agree that we cannot make specific assumptions about the concepts of the tasks and therefore rewrote the paragraph highlighting the different task structure itself and give an example how association between words and food stimuli or between directional movements and food stimuli can oppose each other.

**Comment 16:** *The relationship between BMI and AAT/IAT has been discussed even though only 3 participants had a BMI>30. Since the study finding is not in line with previous findings; other than the 3 participants explanation, there is not much to explain the current finding. The authors could reconsider inclusion of this paragraph? (purely because it does not add any more value to the paper).*

**Response: **The paragraph was removed.

**Comment 17:** *The explanation for the ‘interpersonal differences in the desire to eat individual food items predict approach bias for those individual food items’ appears to be well written as it has well connected with the momentary craving aspect. The effect of valence of food had been correlated with AAT. The discussion does not seem to explain this effect.*

**Response:** We included a sentence that we assume that general liking or disliking of most foods is relatively stable over time and thus we find a relationship with the bias size despite the one-week delay between the picture ratings and bias assessment.

**Comment 18:** *In addition, “it is not uncommon that feature-relevant AATs attain reliability estimates in the upper range across implicit measures (Gawronski et al., 2011) and reliability in current task set-up was lower than in our previous version of the hand-AAT (Kahveci et al., submitted)”. It might be relevant to mention if the reliability was in line with the previous feature-relevant AATs as that might be one of the positive aspects of the study as well.*

**Response:** Unfortunately, it is difficult to compare reliability across feature-relevant studies, as most studies in the food domain do not report reliability and because reliability depends on multiple factors such as stimulus types, task length or variability of true bias scores which differ between studies. Even though reliability of the different task set-ups is not directly comparable when also other factors differ between studies, we put current reliability into perspective by directly citing other feature-relevant AATs.

# Peer review: Reviewer response for version 1

**DOI:** 10.21956/openreseurope.14311.r26592

**Published:** 2021-04-07

**Authors:** Annette Horstmann

**Affiliations:** 1 Department of Psychology and Logopedics, Faculty of Medicine, University of Helsinki, Helsinki, Finland

The authors report on the results of a preregistered study to validate an improved version of the hand-AAT (Approach-Avoidance-Task). In the previous version, effects of block order (congruent vs. incongruent with respect to the target category) were observed and attributed to a low number of blocks, i.e. two. Therefore, the number of blocks was increased from two to six blocks in the present version of the task. Further, the authors aimed to validate the food approach biases measured by the hand-AAT with self-report measures of eating behaviour and an Implicit Association Task. The results indicate reliable food approach biases, mixed results for validity and no observable block order effects in the present version.

 The manuscript is well written and concise. I have a couple of comments that may improve clarity of the report:

1. Eating behaviour surely has a habitual component whose impact may differ on an individual basis. However, it is not correct to describe eating behaviour as purely habitual, and I suggest rephrasing the introductory statement. *“* *Habitual behaviours like eating rely primarily on their implicit association with environmental cues and little on deliberate intentions (* *van't Riet et al., 2011* *).* *”*
2. Please add references for studies that have implemented non-relevant feature / relevant feature AAT in the past.
3. The expectations at the end of the introduction show a certain lack of specific motivation. I would like to invite the authors to elaborate on the motivation/background for each of the very specific expectations.
4. The motivation to use the TEMS is missing in the Methods section. Further, no data or results on this measure are presented and there is no mention in the discussion.
5. The description of the motivational properties of the stimuli could be a bit clearer - in particular, the authors may want to elaborate on the following: Did the authors expect a general food approach bias or a “desired-food” approach bias” only? Did the objects also vary on a comparable scale, i.e., desirability? The IAT included only 12 most liked food items – was validity of the instruments assessed including only those items on the AAT or were all AAT food trials included?
6. The repeated administration of the FCQ-S is not motivated.
7. Regarding the main question of the study, it might be of interest (if possible) to make an explicit assessment of the assumption that the increase in block number led to the absence of block order effects, e.g., is the block order effect still visible when considering the first two blocks only? 

If applicable, is the statistical analysis and its interpretation appropriate?

Yes

Is the study design appropriate and is the work technically sound?

Yes

Is the work clearly and accurately presented and does it engage with the current literature?

Yes

Are the conclusions drawn adequately supported by the results?

Yes

Are sufficient details of methods and analysis provided to allow replication by others?

Partly

Are all the source data and materials underlying the results available?

Yes

Reviewer Expertise:

Cognitive Neuroscience / Experimental Psychology.

I confirm that I have read this submission and believe that I have an appropriate level of expertise to confirm that it is of an acceptable scientific standard, however I have significant reservations, as outlined above.

# Response

**Published:** 2021-06-04

**Authors:** Hannah van Alebeek

**Affiliations:** University of Salzburg, Austria

Dear Prof. Dr. Horstmann,

 On behalf of my co-authors, I would like to thank you for your thorough and detailed review. It helped us to get new insights and we picked up on most of your suggestions. We are especially thankful for guiding us to get a deeper understanding of the remediated block-order effects, which we no longer attribute to the increased number of blocks. Below we outline how we addressed your suggestions:

**Comment 1**: *Eating behaviour surely has a habitual component whose impact may differ on an individual basis. However, it is not correct to describe eating behaviour as purely habitual, and I suggest rephrasing the introductory statement. “Habitual behaviours like eating rely primarily on their implicit association with environmental cues and little on deliberate intentions (van't Riet et al., 2011).”*

**Response:** Based on your comment, we revised the beginning of the paper by indicating that eating behaviour depends on both explicit and implicit processes.

**Comment 2:** *Please add references for studies that have implemented non-relevant feature / relevant feature AAT in the past.*

**Response: **We included some example references during the description of the two instruction types in the second paragraph.

**Comment 3:** *The expectations at the end of the introduction show a certain lack of specific motivation. I would like to invite the authors to elaborate on the motivation/background for each of the very specific expectations.*

**Response:** To support our hypotheses, we revised the last paragraph of the introduction and included additional references linking approach bias to the types of eating behaviours we included in the current study.

**Comment 4:** *The motivation to use the TEMS is missing in the Methods section. Further, no data or results on this measure are presented and there is no mention in the discussion.*

**Response:** The TEMS was included because we speculated, based on results in a previous study, that the current student sample is not representative for the general population with respect to their health and natural concern motive during food choices. We outlined this reasoning in the participant section.

**Comment 5:** *The description of the motivational properties of the stimuli could be a bit clearer - in particular, the authors may want to elaborate on the following: Did the authors expect a general food approach bias or a “desired-food” approach bias” only? Did the objects also vary on a comparable scale, i.e., desirability? The IAT included only 12 most liked food items – was validity of the instruments assessed including only those items on the AAT or were all AAT food trials included?*

**Response:** In the last paragraph of the introduction, we specified that we expect an approach bias to all types of food stimuli as we assume that also less desired foods possess rewarding properties due to their relevance for survival. In the *Materials and apparatus *section, we outlined why we included a wide range of high and low desired foods in the AAT but focussed on highly desired foods in the IAT only and explain our rational for including emotionally neutral objects. In the Results, we specify that we used the same stimuli for the correlation between the AAT and IAT D-scores.

**Comment 6:** *The repeated administration of the FCQ-S is not motivated.*

 Response: In the introduction, we explain that the increase in craving after exposure to food cue can be used to index subjectively perceived cue-reactivity. Thereby motivating the repeated administration of the FCQ-S.

**Comment 7:** *Regarding the main question of the study, it might be of interest (if possible) to make an explicit assessment of the assumption that the increase in block number led to the absence of block order effects, e.g., is the block order effect still visible when considering the first two blocks only?*

**Response:** To investigate the reason for remediated block-order effects, we reanalysed this finding as you suggested. Interestingly, there was no block-order effects when considering the first two or four blocks only. We discuss this new finding in light of possible learning effects.
